# Platinum/Platinum Sulfide on Sulfur-Doped Carbon Nanosheets with Multiple Interfaces toward High Hydrogen Evolution Activity

**DOI:** 10.3390/molecules29194570

**Published:** 2024-09-26

**Authors:** Mou Zhang, Mengfei Su, Chunyan Zhang, Feng Gao, Qingyi Lu

**Affiliations:** 1 State Key Laboratory of Coordination Chemistry, Coordination Chemistry Institute, Collaborative Innovation Center of Advanced Microstructures, School of Chemistry and Chemical Engineering, Nanjing University, Nanjing 210023, China; 2 Jiangsu Key Laboratory of Artificial Functional Materials, Department of Materials Science and Engineering, Collaborative Innovation Center of Advanced Microstructures, College of Engineering and Applied Sciences, Nanjing University, Nanjing 210023, China

**Keywords:** platinum/platinum sulfide, heterogeneous interface, doping, hydrogen evolution reaction

## Abstract

Platinum (Pt)-based materials are among the most competitive electrocatalysts for the hydrogen evolution reaction (HER) due to suitable hydrogen adsorption energy. Due to the rarity of Pt, it is desirable to develop cost-effective Pt-based electrocatalysts with low Pt loading. Herein, Pt/PtS electrocatalysts on S-doped carbon nanofilms (PPS/C) have been successfully fabricated through a precursor reduction route with a complex of Pt and 1-dodecanethiol (1-DDT) as the precursor. The PPS/C achieved at 400 °C (PPS/C-400) exhibits excellent HER performances with an ultralow overpotential of 41.3 mV, a low Tafel slope of 43.1 mV dec^−1^ at a current density of 10 mA cm^−2^, and a long-term stability of 10 h, superior to many recently reported Pt-based HER electrocatalysts. More importantly, PPS/C-400 shows a high mass-specific activity of 0.362 A mg_Pt_^−1^ at 30 mV, which is 1.88 times of that of commercial 20% Pt/C (0.193 A mg_Pt_^−1^). The introduction of sulfur leads to the formation of PtS, which not only reduces the content of Pt but also realizes the interface regulation of Pt/PtS, as well as the doping of carbon. Both regulations make the resulting catalyst have abundant active centers and rapid electron transfer/transport, which is conducive to balancing the adsorption and resolution of intermediate products, and finally achieving great mass-specific activity and stability. The research work may provide ideas for designing effective Pt-based multi-interface electrocatalysts.

## 1. Introduction

Hydrogen energy with a high combustion enthalpy and no carbon dioxide emissions is identified as a sustainable and clean green energy source and attracts extensive attention from academia and industry [1,2,3,4]. Currently, electrocatalytic water splitting represents a crucial area in the hydrogen production industry. Electrocatalysts play key roles during the water splitting, as they enhance the rate, efficiency, and selectivity of the chemical conversion [5,6]. Among the numerous hydrogen evolution electrocatalysts studied, platinum-based (Pt) electrocatalysts remain the most universal choice owing to their optimal interactions with the surface hydrogen adsorbents and can show remarkable performance in the hydrogen evolution reaction (HER) [7,8,9]. However, the scarcity, high price, and unsatisfactory durability of Pt greatly plague its large-scale industrial application [10]. Although non-precious metal-based electrocatalysts have been extensively studied, it is still challenging to find a substitute for platinum due to their low catalytic activities [11,12]. The development of Pt-based electrocatalysts that can meet the various requirements of the HER, including a high catalytic activity, long-term stability, and low loading mass, has been the research focus, and incorporating non-precious elements with platinum as a substitute and dispersing it on support materials without sacrificing performances are expected to improve the utilization efficiency of Pt and reduce the cost.

Recently, a variety of metal/metal sulfide electrocatalysts featuring dual-active centers with heterogeneous interfaces have been successfully employed for hydrogen production, such as Pt/CoS, Pt/WS_2_, Ni/NiS, Ru/MoS_2_, and so forth [13,14,15,16]. The heterogeneous interface is considered as an important region for catalytic reactions. The local electric field generated by a heterogeneous interface can regulate the electronic structure of the active sites, increase the active site number, improve the efficiency of the active sites, promote H_2_ dissociation, and, thus, enhance HER performances [17,18]. Considering the high work function of Pt, it is possible to couple Pt with PtS (Pt/PtS) to accelerate the rapid charge transfer in the electrochemical reaction due to the strong synergistic effect between active Pt and PtS, thus exhibiting excellent HER properties [19]. Beyond that, metal sulfides have many advantages over other catalysts. For example, metal-S ionic bonds are weaker than metal-O bonds, which is more favorable for an electrocatalytic reaction and reduces the catalytic kinetic barrier of electrocatalytic water splitting [20]. In addition, in an acidic medium, the dissolution or aggregation of Pt may affect its HER activity and, thus, result in inferior long-term stability. The addition of PtS would stabilize the active center of Pt by making the available Pt sites more negatively charged, thus becoming more resistant to surface oxidation at a given electrode potential [21,22].

The modification of the support is very important to fix Pt species and achieve appropriate Pt electronic properties, and it is of great significance to select appropriate substrates to improve the utilization efficiency of Pt atoms. Compared to carbon support, carbon support doped with heteroatoms can better anchor Pt and other metals, which helps to enhance the metal-carrier interactions and reduce the aggregation of metal atoms [23,24]. It has been demonstrated that S-doped carbon support with precious metals can help to break the hydrogen–oxygen bonds and reduce the energy barrier of water decomposition [25]. However, the synthesis of sulfur-doped carbon support, the construction of a heterogeneous interface of Pt/PtS, and the loading of hybrid electrocatalyst on the doped support are complex and multi-step processes. To achieve these goals simply will be of great significance for fabricating new electrocatalysts.

Herein, a 1-dodecanethiol (DDT)-assisted precursor method is proposed to fabricate Pt/PtS hybrids with heterogeneous interfaces on S-doped carbon nanofilms (PPS/C). The precursor, hollow Pt-DDT complex nanospheres, is first obtained using a simple solvothermal technique with platinum (II) acetylacetonate as the reactant and DDT and H_2_O as the solvents. The resulting precursor is then calcined in a hydrogen atmosphere. Due to the high temperature instability of Pt-DDT and the reduction characteristics of H_2_, Pt-DDT is reduced to generate Pt nanoparticles or react with the sulfhydryl groups to generate PtS nanoparticles, resulting in the formation of a Pt/PtS heterogeneous structure. In addition, part of sulfhydryl groups reacts with C to form C-S bonds, forming S-doped carbon nanofilms. The realization of heterogeneous interface construction and sulfur doping would introduce a large number of active sites, improve the activity of the active sites, reduce the barrier of the redox reaction, and, thus, result in great performance enhancement. As a result, the appropriate sample PPS/C-400 exhibits excellent HER performances in an acidic electrolyte with an ultralow overpotential of 41.3 mV at a current density of 10 mA cm^−2^. It also shows a great mass-specific activity, which is 1.88 times of that of a commercial Pt/C catalyst. Furthermore, the PPS/C-400 also shows long-term stability in durability tests.

## 2. Results and Discussion

**Figure 1:**
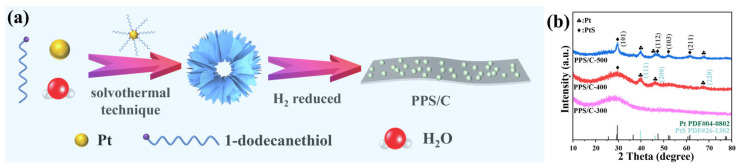
(**a**) Synthesis scheme of the electrocatalyst and (**b**) XRD patterns of the products.

As shown in Figure 1a, the Pt-DDT complex hollow nanospheres are firstly prepared using a solvothermal technique with a reverse microemulsion process. The addition of water to 1-DDT (oil phase) results in the formation of a reverse microemulsion. Under high-temperature solvothermal conditions, the sulfhydryl group of the 1-DDT molecule and the metal Pt of platinum (II) acetylacetonate would combine mightily to form a complex. Due to the soft template effect of the reverse microemulsion, uniform hollow nanospheres composed of nanosheets can be obtained with a diameter of about 3–8 μm, which act as a precursor to be transformed to PPS/C. Due to the low thermal stability of the Pt-DDT complex and the reduction function of H_2_, the precursor is transformed and reconstructed during calcination under a reducing atmosphere (5% H_2_/95% N_2_ mixed gas) with 1-DDT as sulfur and carbon sources. The sulfur source has two functions: on one hand, some S reacts with Pt to form PtS, forming Pt/PtS heterostructures, and on the other hand, the other S combines with C to form C-S bonds, forming S-doped carbon nanofilms, which are obtained from the carbonization of the linear hydrocarbon chain of 1-DDT. Figure 1b shows the XRD patterns of the products obtained at different calcination temperatures. When the calcination temperature is 300 °C, only a broad peak is observed in the XRD pattern (PPS/C-300), indicating that a low temperature is not conductive to the formation of the crystalline phases. With the increase in the annealing temperatures to 400 and 500 °C, intensive diffraction peaks appear. In the XRD pattern of PPS/C-400, the product with an annealing temperature of 400 °C, the diffraction peak at 29.5° belongs to the (101) facets of the tetragonal PtS (PDF#26-1302), while the other diffraction peaks at 2θ = 39.7°, 46.4°, and 67.5° can be indexed to the (111), (200), and (220) crystal facets of the cubic Pt (PDF#04-0802), demonstrating that the Pt-DDT complex precursor has been successfully transformed to Pt/PtS hybrids. When the annealing temperature is further increased to 500 °C (PPS/C-500), more characteristic peaks of PtS can be observed at 36.6°, 47.6°, 52.1°, and 61.7°, indicating that PtS nanoparticles with higher crystallinity have been obtained at the higher annealing temperature.

**Figure 2:**
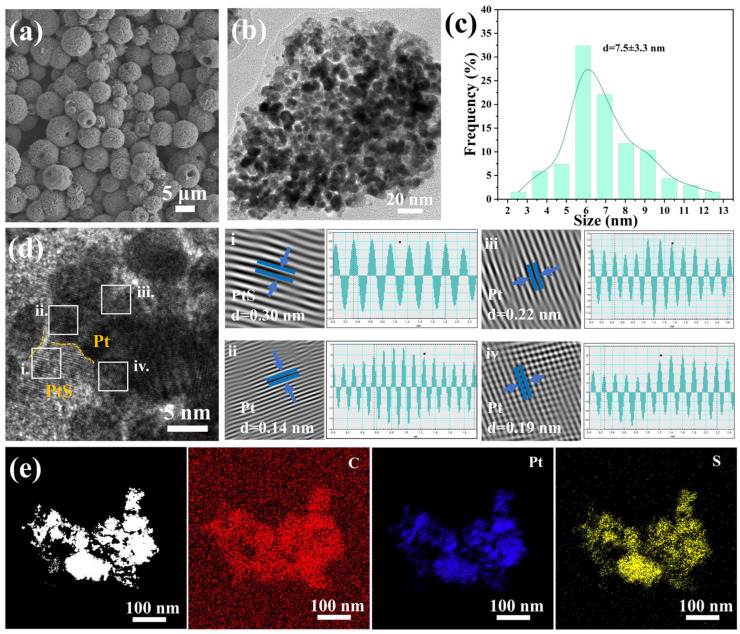
(**a**) SEM image of the Pt-DDT precursor; (**b**,**c**) TEM image and particle size distribution of PPS/C-400; (**d**) HRTEM image and reverse fast Fourier transform images of the corresponding regions of PPS/C-400; (**e**) HAADF-STEM image and EDX element mappings of C, Pt, and S in PPS/C-400.

The morphology and microstructure of the prepared materials were investigated using scanning electron microscopy (SEM) and transmission electron microscopy (TEM). As shown in Figure 2a, the Pt-DDT complex precursor is composed of hollow nanospheres with diameters in the range of 3~8 μm. After calcination, the Pt-DDT complex is transformed to PPS/C. Taking PPS/C-400 as an example, as shown in Figure 2b, due to the low thermal stability of the complex, the resulting PPS/C-400 does not maintain the hollow structure of the complex but instead exhibits a film-like structure with a lot of nanoparticles dispersing. Figure 2c displays the size distribution of the dispersed nanoparticles, revealing that the dispersive Pt/PtS nanoparticles on the carbon film have a diameter ranging from 2 to 13 nm. Figure 2d displays a high-resolution TEM (HRTEM) image of PPS/C-400, from which it can be seen that there are many crystalline lattices with interfaces (some interfaces are marked by bright yellow lines) dispersing on amorphous films. Through inverse fast Fourier transform (IFFT) performed on the regions in the boxes of Figure 2d, the lattice spacings are measured to be 0.30, 0.14, 0.22, and 0.19 nm, which can be indexed to the (101) crystal facets of the tetragonal PtS and the (220), (200), and (111) crystal facets of the cubic Pt, respectively, demonstrating, again, the formation of Pt and PtS hybrids. Figure 2e displays an HAADF-STEM image and corresponding EDX elemental mappings of the PPS/C-400, revealing that C, Pt, and S elements are uniformly distributed in the randomly selected regions. All the above characterization results show that Pt/PtS catalysts supported on S-doped carbon nanosheets have been successfully prepared using the facile precursor method.

**Figure 3:**
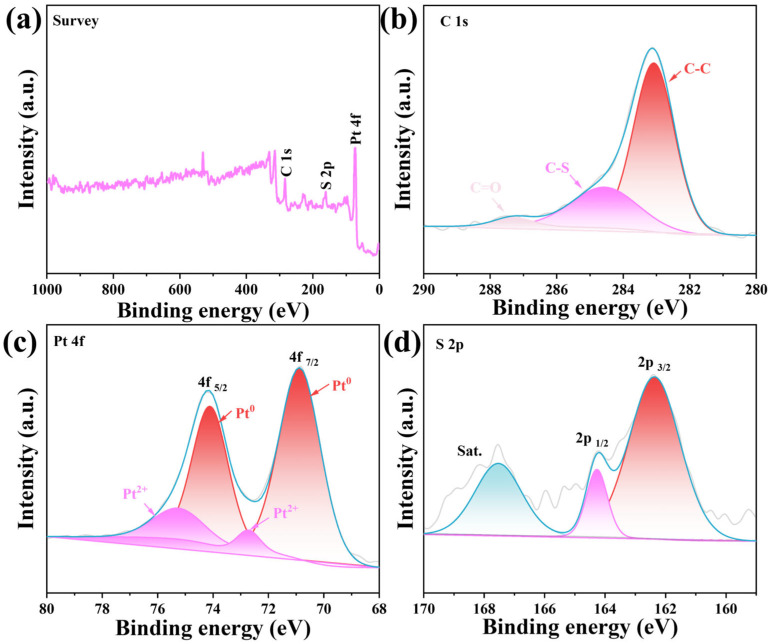
XPS spectra of PPS/C-400: (**a**) survey spectrum; (**b**–**d**) high-resolution XPS spectra of (**b**) C 1s, (**c**) Pt 4f, and (**d**) S 2p.

The surface composition and elemental valence states of PPS/C-400 were further analyzed using X-ray photoelectron spectroscopy (XPS). The XPS survey spectrum in Figure 3a confirms the presence of C, Pt, and S elements in PPS/C-400, which is consistent with the results of the EDX analysis. Figure 3b shows the high-resolution XPS spectrum of C 1s, which can be divided into three peaks belonging to the C-C, C-S, and C=O bonds, respectively, providing a direct evidence for S doping in the carbon films [26]. In Figure 3c, the Pt 4f XPS spectrum demonstrates the presence of metal Pt (70.8 eV and 74.0 eV) and Pt^2+^ (72.7 eV and 75.2 eV) [27,28]. In the S 2p XPS spectrum (Figure 3d), the peaks with the binding energies of 162.3 and 164.2 eV are attributed to 2p_3/2_ and 2p_1/2_ of S, respectively, while that at 167.5 eV is attributed to the corresponding satellite peaks (labelled as Sat.) [29]. XPS analyses further confirm the formation of Pt/PtS on S-doped carbon.

**Figure 4:**
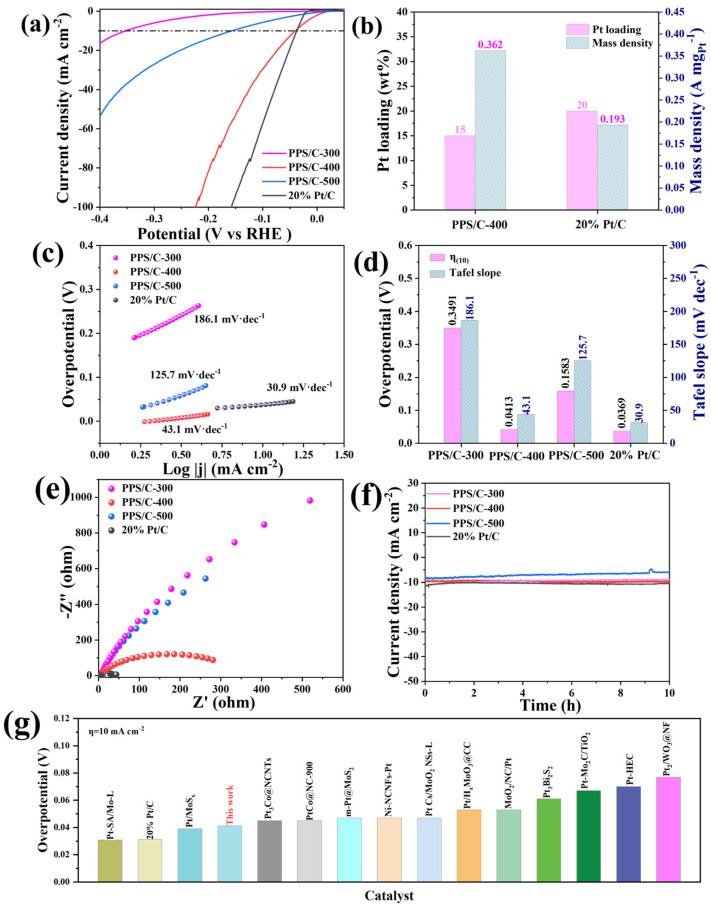
Electrochemical HER performances of the electrocatalyst in 0.5 M H_2_SO_4_ electrolyte: (**a**) LSV curves; (**b**) comparison of mass-specific activities of PPS/C-400 and commercial Pt/C at 30 mV; (**c**) Tafel slopes; (**d**) overpotential and Tafel slope values at a current density of 10 mA cm^−2^; (**e**) Nyquist curves; (**f**) I-t curves of the PPS/C-300, PPS/C-400, PPS/C-500, and commercial Pt/C electrocatalysts; (**g**) comparison of the overpotentials between PPS/C-400 and recently reported Pt-based electrocatalysts at 10 mA cm^−2^.

The HER performances of the obtained electrocatalysts were evaluated using a standard three-electrode system in a 0.5 M H_2_SO_4_ electrolyte at room temperature. Linear sweep voltammetry curves (LSV) were first collected at a sweep rate of 5 mV s^−1^. As shown in Figure 4a, among the synthesized electrocatalysts, PPS/C-400 exhibits the best HER performances with the lowest overpotential of 41.3 mV at a current density of 10 mA cm^−2^, which is close to that of 36.9 mV achieved using commercial Pt/C (20 wt%), reflecting excellent HER activity of the Pt/PtS catalysts supported on S-doped carbon nanofilms. The overpotential of PPS/C-500 at a current density of 10 mA cm^−2^ is 158.3 mV, indicating that the overgeneration of PtS may not be conducive to the improvement in HER performance. In addition, PPS/C-300 requires a large overpotential of 349.1 mV to drive the current density of 10 mA cm^−2^, indicating its poor HER performance, which may be due to the fact that it is difficult to reduce the Pt element from the precursor at 300 °C. Figure 4b shows the mass percentage of Pt quantified from XPS analyses, revealing that the mass percentage of Pt in PPS/C-400 is about 15 wt%, which is lower than the 20 wt% of the commercial Pt/C. Importantly, with the low loading of Pt, under the overpotential of 30 mV, the mass activity value of the PPS/C-400 can achieve to be 0.362 A mg_Pt_^−1^, which is 1.88 times of that of commercial 20 wt% Pt/C (0.193 A mg_Pt_^−1^). Figure 4c,d presents the Tafel slopes of PPS/C-300, PPS/C-400, PPS/C-500, and commercial Pt/C to reflect the adsorption and desorption kinetics during the HER. Among them, PPS/C-400 has a slope value of 43.1 mV dec^−1^, which is smaller than that of PPS/C-300 (186.1 mV dec^−1^) and PPS/C-500 (125.7 mV dec^−1^), indicating that it has the fastest reaction kinetics among the synthesized catalysts. The HER kinetic behavior of PPS/C-400 is likely affected by the Volmer–Heyrovsky mechanism because the slope value is in the range of 40–120 mV dec^−1^ [30,31]. A low overpotential indicates that less energy is required for electrocatalysis, while a low Tafel slope signifies enhanced reaction kinetics in electrochemical processes. Both the low overpotential and low Tafel slope demonstrate that the synthesized electrocatalyst PPS/C-400 exhibits excellent HER catalytic performances. Electrical impedance spectroscopy (EIS) is another index that can reflect the kinetic speed of the electrocatalysis. As shown in Figure 4e, PPS/C-400 has a lower charge transfer resistance than PPS/C-300 and PPS/C-500, demonstrating its high electrochemical intrinsic activity. Long-term electrocatalytic stability is also an important index to evaluate the HER performances of the catalytic materials. As shown in Figure 4f, after 10 h of the operation, the voltage to drive the current density of 10 mA cm^−2^ for the prepared catalysts basically does not attenuate, indicating that the prepared electrocatalysts with multiple interfaces can be used as a highly stable electrocatalyst for the HER, which may be attributed to the protection of PtS against the surface oxidation of Pt and the synergistic multi-interface effect in the HER process. A comparison of PPS/C-400 with some of the representative Pt-based HER electrocatalysts is shown in Figure 4g and Table 1, exhibiting that the prepared PPS/C-400 with multiple interfaces has a similar or better activity than these catalysts. The results above show that PPS/C-400 with multiple interfaces has been successfully constructed using a facile DDT-assisted precursor method, and the synergistic multi-interface effect of Pt/PtS, the S-doping on carbon, and the carbon–metal support plays important roles in the excellent catalytic performances of the HER process. The introduction of sulfur leads to the formation of PtS and successfully reduces the content of Pt to 15 wt%, resulting in its superior mass activity to commercial Pt/C catalyst. The lower loading of Pt combined with the high mass activity will undoubtedly lower the catalyst’s cost, thereby enhancing its application potential in practical scenarios. Furthermore, the formation of PtS also realizes the interface construction of Pt/PtS, resulting in abundant active centers and rapid electron transfer/transport, which is conducive to balancing the adsorption and resolution of the intermediate products and finally achieving higher mass-specific activity and stability.

## 3. Experimental

### 3.1. Synthesis of Pt-1-Dodecanethiol Hollow Nanospheres

The Pt-DDT complex precursor was prepared using a solvothermal technique with platinum (II) acetylacetonate as the reactant and 1-dodecanethiol (DDT) and H_2_O as the solvents. Initially, 20 mg of platinum (II) acetylacetonate was dissolved in a mixture of 5 mL DDT and 1 mL H_2_O. After the mixture was stirred vigorously for a certain time to form a stable dispersion, it was transferred into a 50 mL Teflon-lined stainless-steel autoclave and kept at 160 °C for 12 h. After cooling to room temperature, the precursor was collected through centrifuging, washed with ethanol and deionized water several times, and dried at room temperature. The obtained Pt-1-dodecanethiol complex precursor is named Pt-DDT.

### 3.2. Synthesis of Pt/PtS/C

To synthesize the Pt/PtS/C electrocatalyst, a certain amount of Pt-DDT was added in a porcelain boat, which was placed in a tube furnace. Subsequently, the tube furnace was heated to the set temperatures with a heating rate of 5 °C min^−1^ and kept for 2 h under an atmosphere of 95% N_2_/5% H_2_. The temperatures were set as 300, 400, and 500 °C, and the corresponding products were named as PPS/C-300, PPS/C-400, and PPS/C-500, respectively.

### 3.3. Characterizations

X-ray diffraction (XRD) patterns of the products were obtained using a Bruker D8 ADVANCE diffractometer with Cu K_α_ radiation (XRD, BRUKER, Karlsruhe, Germany). A Hitachi S-4800 scanning electron microscope (SEM, HITACHI, Tokyo, Japan) was used to collect the morphology characteristics of the product at an acceleration voltage of 5 kV. Transmission electron microscopy (TEM, JEOL, Akishima, Japan) images and element mappings were collected using a JEM-2800 Plus transmission electron microscope with an electron acceleration voltage of 200 kV. X-ray photoelectron spectroscopy (XPS, PHI 5000 reverse probe type, ULVAC-PHI, Chigasaki, Japan) was used to characterize the chemical environment of the elements of the samples.

### 3.4. Electrochemical Measurements

Electrochemical tests were performed at room temperature using a conventional three-electrode system on the electrochemical workstation CHI 660E (Chenhua Instruments Co., Ltd., Shanghai, China). A glass carbon electrode with catalyst loading was used as the working electrode, while a graphite carbon rod and Ag/AgCl were used as the opposite electrode and reference electrode respectively. A total of 3.0 mg of as-prepared catalyst was dispersed in a mixture of 0.15 mL of H_2_O, 0.05 mL of isopropyl alcohol, and 0.01 mL of Nifion (5 wt%) to form a uniform ink. The glassy carbon electrode was polished with Al_2_O_3_ powder (0.3 and 0.05 μm), thoroughly rinsed with deionized water, and dried. Then, 10 μL of catalyst ink was carefully dropped onto the surface of the glass carbon electrode and dried by air. The linear sweep voltammetry polarization curve (LSV) was recorded at a 5 mV s^−1^ scanning rate. Electrochemical impedance spectroscopy (EIS) was performed at frequencies ranging from 100,000 to 0.1 Hz. Amperometric current–time measurements (I-t) were collected for 10 h at a fixed current density of 10 mA cm^−2^. The measured potential was expressed using the Nernst equation and converted into a reversible hydrogen electrode potential.

## 4. Conclusions

In summary, we prepared an efficient HER electrocatalyst consisting of Pt/PtS on S-doped carbon nanosheets with a low loading of Pt and multiple interfaces through heat treatment of a Pt-DDT precursor. The dissociated S from the sulfhydryl group of DDT reacts with Pt to form PtS, which can reduce the amount of Pt and form a Pt/PtS heterojunction; it can be also doped into carbon nanosheets to form the S-doped support. The resulting PPS/C-400 achieves a low overpotential of 41.3 mV and a Tafel slope of 43.1 mV dec^−1^ at a current density of 10 mA cm^−2^ in 0.5 M H_2_SO_4_, which is superior to many recently reported Pt-based HER electrocatalysts, and remains stable under long-term testing. The mass activity of PPS/C-400 at 30 mV is 1.88 times of that of commercial Pt/C, demonstrating its efficient use of Pt. The enhanced catalytic activity and stability of PPS/C-400 are strongly correlated with the synergistic multi-interface effect of the interactions of Pt/PtS and S-doped carbon nanosheets in the HER process, which not only provide abundant active sites and rapid electron transfer ability but are also conducive to the adsorption and dissociation of intermediate products. This work illustrates the benefits of multi-interface electrocatalysts for the HER and provides guidance for the design of low-loading Pt-based electrocatalysts with multiple interfaces.

## Figures and Tables

**Table 1:** HER performance comparisons of PPS/C-400 with recently reported Pt-based electrocatalysts at a current density of 10 mA cm^−2^ in 0.5 M H_2_SO_4_.

Catalysts	Overpotential (mV)	Reference
Pt-SA/Mo-L	31	*Adv. Mater.* **2024**, *36*, 2305375 [32]
20% Pt/C	36.9	/
Pt-MoS_x_	39	*J. Phys. Chem. C* **2024**, *128,* 7483–7495 [33]
**PPS/C-400**	**41**	This work
Pt_3_Co@NCNTs	45	*ACS Appl. Mater. Interfaces* **2024**, *16*, 520–529 [34]
PtCo@NC-900	45	*J. Phys. Chem. Lett.* **2022**, *13*, 5195–5203 [35]
m-Pt@MoS_2_	47	*Small* **2024**, *20*, 2309427 [36]
Ni-NCNFs-Pt	47	*J. Colloid Interface Sci.* **2018**, *514*, 199–207 [37]
Pt Cs/MoO_2_ NSs-L	47	*Nano Energy* **2019**, *62*, 127–135 [38]
Pt/H_x_MoO_3_@CC	53	*Int. J. Hydrogen Energy* **2024**, *51*, 701–708 [39]
MoO_2_/NC/Pt	53	*ACS Appl. Nano Mater.* **2024**, *7*, 17364–17372 [40]
Pt_3_Bi_2_S_2_	61	*Chem. Commun.* **2021**, *57*, 7946–7949 [41]
Pt-Mo_2_C/TiO_2_	67	*Appl. Surface Sci.* **2020**, *509*, 144679 [42]
Pt-HEC	70	*Adv. Mater. Tech.* **2023**, *8*, 2200882 [43]
Pt_2_/WO_3_@NF	77	*Electrochim. Acta* **2024**, *475*, 143599 [44]

## Data Availability

No new data were created except for the data in the paper.
